# TR Alpha 2 Exerts Dominant Negative Effects on Hypothalamic *Trh* Transcription *In Vivo*

**DOI:** 10.1371/journal.pone.0095064

**Published:** 2014-04-18

**Authors:** Hajer Guissouma, Rym Ghaddab-Zroud, Isabelle Seugnet, Stéphanie Decherf, Barbara Demeneix, Marie-Stéphanie Clerget-Froidevaux

**Affiliations:** 1 Laboratoire de Génétique, Immunologie et Pathologies Humaines, Département de Biologie, Faculté des Sciences de Tunis, CAMPUS, Université Tunis El Manar, Tunis, Tunisie; 2 UMR CNRS 7221, Evolution des Régulations Endocriniennes, Department Régulations, Développement et Diversité Moléculaire, Muséum National d'Histoire Naturelle, Paris, France; University Claude Bernard Lyon 1, France

## Abstract

Mammalian thyroid hormone receptors (TRs) have multiple isoforms, including the *bona fide* receptors that bind T_3_ (TRα1, TRβ1 and TRβ2) and a non-hormone-binding variant, TRα2. Intriguingly, TRα2 is strongly expressed in the brain, where its mRNA levels exceed those of functional TRs. Ablation of TRα2 in mice results in over-expression of TRα1, and a complex phenotype with low levels of free T_3_ and T_4_, without elevated TSH levels, suggesting an alteration in the negative feedback at the hypothalamic-pituitary level. As the hypothesis of a potential TRH response defect has never been tested, we explored the functional role of TRα2 in negative feedback on transcription of hypothalamic thyrotropin, *Trh*. The *in vivo* transcriptional effects of TRα2 on hypothalamic *Trh* were analysed using an *in vivo* reporter gene approach. Effects on *Trh-luc* expression were examined to that of two, T_3_ positively regulated genes used as controls. Applying *in vivo* gene transfer showed that TRα2 over-expression in the mouse hypothαlamus abrogates T_3_-dependent repression of *Trh* and T_3_ activation of positively regulated promoters, blocking their physiological regulation. Surprisingly, loss of function studies carried out by introducing a shTRα2 construct in the hypothalamus also blocked physiological T_3_ dependent regulation. Thus, modulating hypothalamic TRα2 expression by either gain or loss of function abrogated T_3_ dependent regulation of *Trh* transcription, producing constant transcriptional levels insensitive to feedback. This loss of physiological regulation was reflected at the level of the endogenous *Trh* gene, were gain or loss of function held mRNA levels constant. These results reveal the as yet undescribed dominant negative role of TRα2 over TRα1 effect on hypothalamic *Trh* transcription.

## Introduction

Thyroid hormone (TH) production is controlled by the hypothalamic peptide Thyrotropin Releasing Hormone (TRH). T_3_ exerts negative feedback on *Trh* transcription mainly through the beta forms of the thyroid receptors (TRβ1 and TRβ2) [1], [2]. TRs are ligand-dependent transcription factors [3], produced from two genes: NR1A1 and NR1A2 [4], [5]. Each gene gives rise to two major isoforms, respectively TRα1 and α2, and TRβ1 and β2, by alternative splicing. Both RNA [6], [7] and protein [8], [9] for each isoform are found in the hypothalamic paraventricular nucleus (PVN), site of *TRH* regulation.

In mammals, TRα2 is identical to TRα1 in its N-terminus, but the C-terminus is entirely different rendering TRα2 unable to bind T_3_ [10], [11] and altering the ability of TRα2 to interact with co-activators and co-repressors [12], [13]. As TRα2 can bind DNA, but not activate transcription, it has been suggested that TRα2 may act as a dominant-negative receptor. *In vitro*, TRα2 blocks the activity of other TRs by competing for TR binding to thyroid hormone response elements (TREs) on DNA [14]–[16] or *via* mechanisms that do not require TRE binding [17]. TRα2 is widely expressed and in brain, its RNA levels greatly exceed those of the functional TRs, especially in perinatal period [18]. Moreover, TRα2 is highly conserved in human, rat and mouse, but is absent in non mammalian vertebrates [19], suggesting an important function for this protein in mammals.

Generation of mutant mice lacking TRα2 has contributed to understand the roles of TRα2 on T_3_-dependent regulation of target genes in the brain [20]. In these TRα2^-/-^mice, TRα2 ablation results in TRα1 over-expression in brain tissue, and lower levels of free T_3_ and T_4_ but normal levels of TSH. This failure of TSH to adjust to the lower circulating T_3_ and T_4_ levels can be accounted for either by an effect at the level of the thyroid gland reducing hormone production, and/or an alteration in the negative feedback at the hypothalamic-pituitary level, which may also include a defect in TRH response. However, this latter hypothesis has never been tested. Previous studies on TRα2 function in brain have attributed a general dominant negative effect of TRα2 but never addressed its transcriptional effects on target genes *in vivo*, because of the technical challenge it represents. Thus we employed a synthetic gene transfer method in which our laboratory has a great expertise to follow the effects of TRα2 gain or loss of function on *Trh* gene transcription using positively regulated T_3_ genes (Malic and Tyrosine hydroxylase Enzymes; respectively, *ME* and *TyrH*) as controls. This *in vivo* transfection assay provides for tissue specific physiological regulation of transcription in integrated contexts [1], [2]. We used the newborn mouse brain as a model system as it was successfully used to analyse the molecular basis of thyroid hormone dependent effects of *Trh* transcription *in vivo* [1], [2], [21] and mainly because that every transcriptional regulation we have identified by this method has later been ratified by experiments in adult transgenic mice.

*In vivo* over-expression experiments show that in the hypothalamus, TRα2 acts as a dominant-negative receptor, blocking transcription of both positively and negatively T_3_ regulated target genes. Moreover, transient TRα2 knockdown seems to reveal TRα1 effect on *Trh* promoter, the regulation of which being equivalent to the one observed when TRα1 is over-expressed. This hypothesis was emphasised by a decrease in circulating T_4_ following TRα1 gain of function. Interestingly, both gain or loss of TRα2 function seems to block *Trh* transcription at an intermediate level between activated and repressed control levels. Indeed, an average TRH activity remains, whereas fine physiological T_3_ regulation is lost. Taken together, these results reveal the physiological importance of TRα2, naturally acting as dominant-negative receptor on hypothalamic *Trh* transcription *in vivo*.

## Materials and Methods

### Ethics Statement

All aspects of animal care and experimentation were in accordance with the National institutes of Health Guidelines for the Care and Use of Laboratory Animals and approved by the Institutional Animal Care and Use Committee of the Animal Protection and Health, Veterinary Services Direction, Paris, France.

### Animals

Swiss wild-type mice were purchased from Janvier (Le Genest St. Isle, France). To induce foetal and neonatal hypothyroidism, dams were given iodine-deficient food containing 0.15% 6-n-propyl-2-thiouracil (PTU) (Harlan) and drinking water with 0.5 g/l PTU (Sigma-Aldrich) from day 14 of gestation through lactation.

### Plasmids

*Trh-luciferase (Trh-luc)* and rat (r) pSG5-TRα1 constructs have been described previously [21]. *Tyrosine hydroxylase-luciferase* reporter plasmid (*TyrH-luc*), containing 800 pb of the rat *Tyrosine hydroxylase* gene promoter [22] cloned upstream the *firefly luciferase* coding sequence in the pGL2 backbone, was a gift from Dr J Mallet (UMR 7091, Hôpital de la Pitié-Salpêtrière, Paris, France). Human (h) TRα2 in pSG5 was kindly provided by Dr. Chassande and Dr Samarut (ENS de Lyon, France). TRα2 is highly conserved in man, rat and mouse.

To knockdown endogenous TRα2, shRNA-coding plasmids were designed against TRα2 (CMV H1- shTRα2), providing two shRNA sets (sh1TRα2 and sh2TRα2) as described in Decherf et al. ([23], Supporting Information). Each shRNA-coding sequence was purchased from Eurogentec. The control plasmid used was CMV-H1 ([23], SI). The shTRα2 contains sense and antisense siRNA sequences, as following:

For sh1TRα2, siRNA sequences are: sense (5′- AAGGACAGCAGCTTCTCGGATT-3′) and antisense (5′- AATCCGAGAAGCTGCTGTCCTT-3′)

For sh2TRα2, siRNA sequences are: sense (5′- TGCAGAGTTCGATTCTGTACTT-3′) and antisense (5′- AAGTACAGAATCGAACTCTGCA-3′).

### *In Vivo* Gene Transfer (*iGT*) and Luciferase Assays

DNA/PEI (polyethylenimine) complexes, *iGT* and luciferase assays were carried out as described previously [1]. Given the highly tissue-specific nature of *Trh* transcription, one of the most important steps in ensuring reproducibility is careful and consistent injection, followed by precise dissection of the hypothalamic areas transfected [1]. Briefly, pups were anesthetized by hypothermia on ice and transfected on post-natal day 2. A glass micropipette was lowered 2 mm through the skull, 0.5 mm posterior to bregma on the sagittal suture, into the hypothalamic area. Two-day-old hypothyroid newborn mice were transfected in the hypothalamic region of the brain with 2×2 µL of *Trh-luc*, or *ME-tk-luc* or *TyrH-luc* (1 µg/pup) complexed with PEI. To assess the effect of TR overexpression, in addition of the reporter genes, we added pSG5-TRα1, pSG5-TRα2, or empty pSG5 expression vector in the complexes at 100 ng/pup. Luciferase activity was assayed 18 h after transfection. In shRNA experiments, we added small hairpin expression vector (see section plasmids) at a 100 ng/µL concentration (400 ng/pup). After 48 h, pups were decapitated, and hypothalami were dissected out for luciferase assays following the manufacturer's protocol (Promega). Luciferase activity was measured 48 h later to allow for shRNA expression.

For qPCR analysis, pups were only transfected with either the overexpression vectors or small-hairpin RNA vectors. Transfections were performed in 2 days old pups and the hypothalami were dissected at 1, 3 and 5 days post-transfection for overexpression experiments, and at 36 h post transfection (3.5 days) for sh experiments.

### Animal treatments

To assess T_3_ effects on reporter gene expression, pups were injected subcutaneously, with 2.5 µg/g of body weight (bw) of T_3_ (Sigma-Aldrich, St Quentin Fallavier, France) in 0.9% saline, immediately after transfection. This quite high dose of T_3_ is necessary to observe *Trh* gene regulation in the hypothalamus of newborn mice, because global metabolic rate is high at this developmental stage and the brain is a resistant organ to excess of TH levels [7], [24]. Controls received the same volume of 0.9% saline. In the shRNA experiments, this procedure was repeated 24 h after transfection.

### Measurement of total plasma T_4_

Frozen plasma was thawed and processed according to the supplier's instructions, using the AMERLEX-M T_4_ RIA Kit (Trinity Biotech, Wicklow, Ireland). Results are expressed as means ± SEM.

### Immunoblot analysis

Western blot analysis was made on hypothalamus protein extract from brains transfected with CMV H1-shTRα2. Briefly, the hypothalami of two distinct mice were collected for each group under a stereo-microscope. The whole experiment was repeated twice. Tissues were lysed mechanically and proteins were extracted in RIPA buffer (150 mM NaCl, 1% Triton X-100, 0.5% Sodium deoxycholate, 0.1% SDS, 50 mM Tris pH 8) according to the manufacturer's instructions. Protein content was determined by Qbit assays (Invitrogen). Total cell lysates (30 µg) were fractionated by SDS-PAGE 4–20% (Pierce) and transferred to nitrocellulose membranes (Biorad). Membranes were blocked with 5% non fat milk in Tris-buffer saline (TBS; 10 mM Tris–HCl, pH 7.5, 150 mM NaCl), followed by overnight incubation at 4°C with the indicated antibody diluted in TBS with 0.05% Tween-20 (TBS-T). After three washes with TBS-T, membranes were incubated with the appropriate secondary antibody coupled to peroxidase, and immunocomplexes visualized by enhanced chemiluminescence (ECL plus from GE Healthcare Amersham) according to manufacturer's instructions. Primary antibodies for Western-blotting, included rabbit polyclonal anti-TRα2 (1∶100; Millipore), rabbit polyclonal anti-βACTIN (1∶3000; Sigma). Secondary antibody was anti-rabbit IgG Peroxidase Conjugate from Sigma. Chemiluminescence was revealed by film exposure.

### RNA extraction and cDNA synthesis

Hypothalami were dissected from individual newborn mice (transfected either by overexpression vector or small-hairpin RNA vector (see section *in vivo* gene transfer) under stereo-microscope (limits for hypothalamic dissection: posterior to the optic chiasma, anterior to the mammillary bodies, along both lateral sulcus and 1 mm in depth) and kept in “RNAlater” (Ambion Inc, Austin, TX, USA) until extraction. RNA extraction was performed using RNAble reagent following manufacturer's protocol (Eurobio, Les Ulis, France). Concentration (A260) of the total RNA was determined and RNA was stored in Tris 10 mM/EDTA 0.1 mM (PH 7.4) at −80°C.

Prior to qPCR, 2 µg of total RNA were reverse-transcribed using Superscript II Rnase H-reverse transcriptase (Invitrogen, Carlsbad, CA, USA) according to manufacturer's protocol. Control reactions without reverse-transcriptase were done in parallel.

### Primers

18S primers and TaqMan probe were provided in the kit Eukaryotic 18S rRNA Endogenous Control (VIC/MGB Probe, Primer Limited) from Applied Biosystems, Warrington, UK. *Trh* primers were described in [7].

### Quantitative polymerase chain reaction (QPCR)

Direct detection of the PCR product was monitored by measuring the increase in fluorescence generated by the TaqMan probe (18S) or by the binding of SYBR Green to dsDNA (*Trh*). For *Trh*, 2 µl of cDNA were added to a mix containing *Trh* primers (300 nM), and 2x SYBR Green Master Mix (Applied Biosystems) to a final reactional volume of 20 µl. For 18S RNA (endogenous control), samples containing 2 µl of cDNA, 1 µl of 18S probe and 10 µl of 2x TaqMan^R^ universal PCR Master Mix (Applied Biosystems) were prepared in a final volume of 20 µl. The gene-specific PCR products were measured continuously by means of ABI PRISM 7300 Sequence Detection System (Applied Biosystems) during 40 cycles. All experiments were run in duplicate, and the same thermal cycling parameters were used (95°C for 10 min (1 cycle), 95°C for 15 sec and 60°C for 1 min (40 cycles)). Non-template controls and dissociation curves were used to detect primer-dimer conformation and non-specific amplification. According to the widely accepted MiQE (Minimum Information for Publication of Quantitative Real-Time PCR Experiments, [25]) guidelines, we verified the efficiency of the PCR for *trh* set of primers by using a serial 10 times dilution of the template. The dynamic range covered four orders of magnitude. We determined amplification efficiency from the slope of the log-linear portion of the calibration curve. The resulting efficiency was close to 100% (97%). Given this efficiency, which is required to be able to use the ddCT relative quantification method, we can deduce that we can use these primers for standard quality qPCR studies. The threshold cycle (CT) of each target product was determined and ΔCT between target and endogenous control was calculated. The CT is the number of PCR cycles required for the fluorescence signal to exceed the detection threshold value. The detection threshold was set to the log linear range of the amplification curve and kept constant for all data analysis. The difference in ΔCT values of two genes (ΔΔCT) was used to calculate the fold difference (F = 2^-ΔΔCT^). The relative quantitative results were used to determine changes in *Trh* gene expression in groups where TRα1 or TRα2 was overexpressed as compared to control samples (empty pSG5 vector) at the ages shown. In the shRNA experiments, the *Gapdh* [23] was used as an endogenous control gene for normalisation.

### Statistical analysis of the results

For *in vivo* gene transfer, results were expressed as the mean ± SEM from an appropriate number of experiments as indicated in the figure legends. Nonparametric test with permutations (*StatXact* Cytel Studio software, Cambridge, MA) was used to assess for statistical differences. For post-test comparisons, we took into account the multiple testing factor, using a non parametric solution. p<0.05 was considered significant (*, p<0.05; **, p<0.01; ***, p<0.001). Each experiment was carried out with n ≥10, repeated at least two times providing the same results. For qPCR experiments, data were plotted as traditional Tukey whiskers (represent 1.5 times the interquartile distance or to the highest or lowest point, whichever is shorter). Statistical analysis compared the median of ΔCT values using nonparametric ANOVA, followed by a permutation test (*StatXact* Cytel Studio software, Cambridge, MA) to compare the control and treated groups.

## Results

### TRα2 exerts dominant negative activity on positively and negatively regulated T_3_ target genes in the mouse hypothalamus *in vivo*

To test whether TRα2 acts as a dominant-negative receptor on hypothalamic gene transcription *in vivo*, two reporter gene assays were carried out, using a positively T_3_ regulated promoter (*ME-tk-luc*) and a negatively regulated one (*Trh-luc*). In both cases we compared the effects of TRα2 over-expression to those of TRα1 overexpression, the action of which being already well characterised on both promoters *in vivo*.

**Figure 1:**
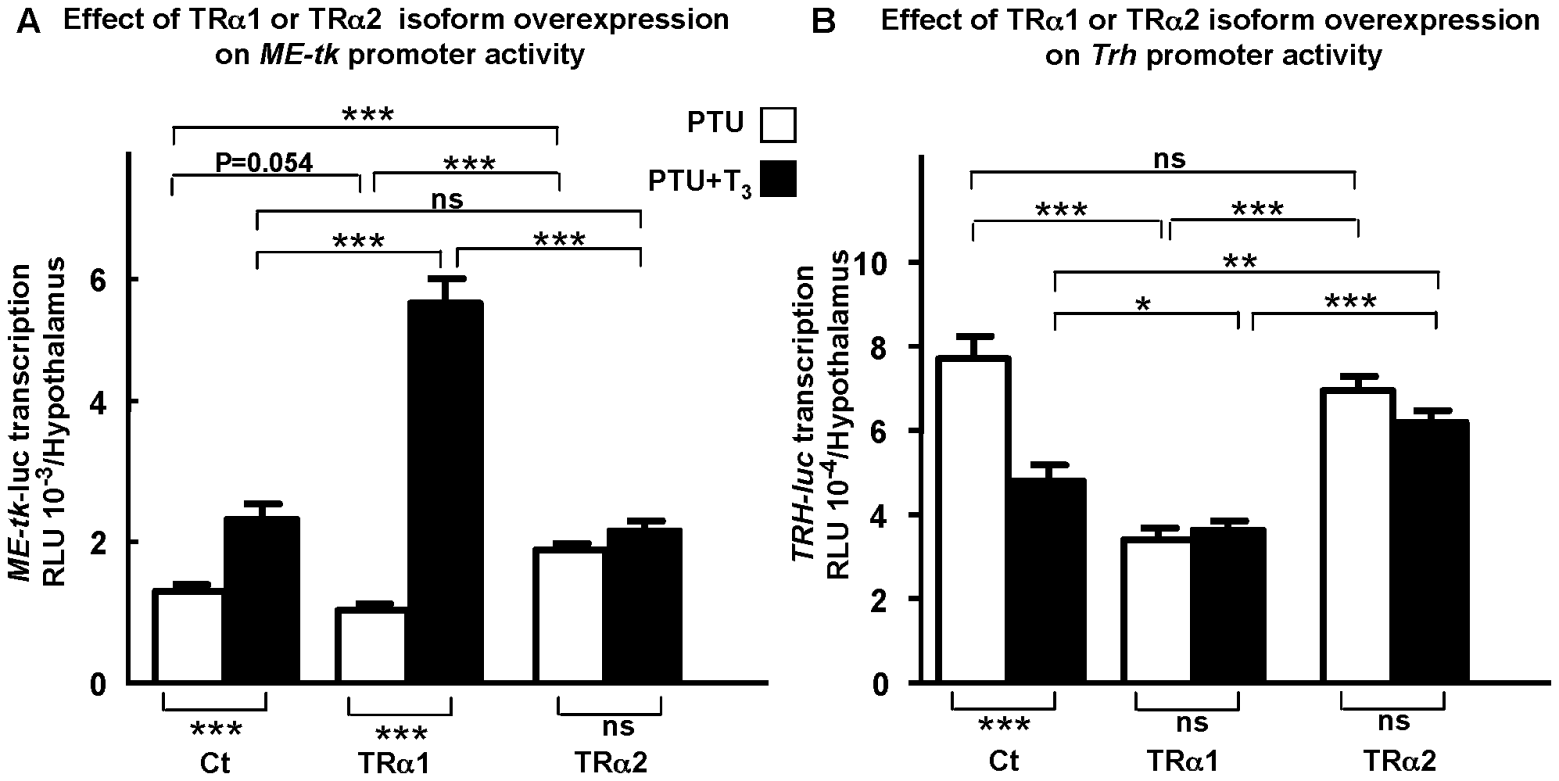
TRα2 shows dominant negative activity on positively and negatively regulated T_3_ target genes *in vivo*. **A**: **TRα2 exerts dominant negative activity on positively** ***ME-tk-luc*** **transcription.** *ME-tk-luc* transcription was measured in hypothyroid (PTU) 2 days old mice treated with T_3_ (2.5 µg/g b.w.) (PTU+T3) or saline (PTU), 18 h after hypothalamic injection of 1 µg reporter construct and 100 ng expression vector (empty pSG5 (Ct) or pSG5-TRα1 (TRα1) or pSG5-TRα2 (TRα2)). Transcription from *ME-tk-luc* is significantly increased in the presence of T_3_ when TRα1 is overexpressed (as compared to Ct) (p<0.001). In contrast TRα2 overexpression significantly increases basal, T_3_-independent *ME* transcription as compared to Ct and TRα1 (p<0.001), but addition of T_3_ does not modify transcription further. **B**: **TRα2 exerts dominant negative activity on negatively** ***Trh-luc*** **transcription.** *Trh-luc* transcription was measured in hypothyroid (PTU) 2 days old mice as described above (100 ng expression vector and 1 µg reporter gene, *Trh-luc* per pup). Transcription from a *Trh-luc* construct is significantly decreased both in absence (PTU) and presence of T_3_ (PTU+T3) when TRα1 is overexpressed (as compared with Ct). In contrast, overexpression of TRα2 has no effect on T_3_-independent *Trh* transcription, but blocks its T_3_-dependent repression. SEMs are given, n≥10 per point. In each case, the whole experiment was repeated twice giving similar results. *, p<0.05; **, p<0.01; ***, p<0.001.

When using the *ME-tk-luc* construct in the *in vivo* transfection paradigm, we found that in controls (figure 1A, left columns), T_3_ significantly increased *ME-tk-luc* transcription by two fold (p<0.001). When TRα2 was co-transfected with *ME-tk-luc*, transcription was blocked at the basal level seen in controls in the presence of T_3_ (figure 1A, far right columns). Thus, TRα2 over-expression blocks the stimulatory effect of endogenous receptors on *ME-tk-luc* transcription. In contrast, co-transfection of TRα1 activated *ME-tk-luc* transcription about five fold in the presence of T_3_ (figure 1A, middle pair of columns, p<0.001), but did not modify transcription levels in absence of T_3_ (p = 0.054). Thus, TRα2 does act as a dominant-negative receptor *in vivo*, blocking the regulation of transcription from a positively regulated TRE.

We next examined the effects of TRα2 on the negatively regulated *Trh* gene, using the same *in vivo* gene transfer paradigm. In control mice, expression from the *Trh-luc* construct (co-transfected with an empty expression vector) was reduced by 37% in animals injected with T_3_ as compared with animals receiving saline (first pair of columns in figure 1B). This repression was significant with a p value <0.001. When the TRα2 isoform was over-expressed, it abolished physiological regulation of *Trh*. As expected, given that TRα2 cannot bind ligand, T_3_ had no effect on transcription levels induced by TRα2. However, in both cases (TRα2 with or without T_3_ (figure 1B, far right columns) *Trh-luc* levels were raised to the maximum levels seen in controls (i.e. in activated, saline injected hypothyroid animals, figure 1B, left columns). Thus, here again TRα2 was acting as a dominant-negative receptor, blocking the effects of the functional endogenous TRs. As an internal control, we used TRα1 which is known to inhibit both T_3_-dependent and T_3_-independent regulation of *Trh* [21]. As expected, TRα1 blocked *Trh* transcription at low levels, at 55% of the T_3_-independent control level whether or not T_3_ was present.

### Transient TRα2 knockdown has no transcriptional effect on positively and negatively regulated T_3_ target genes *in vivo*

**Figure 2:**
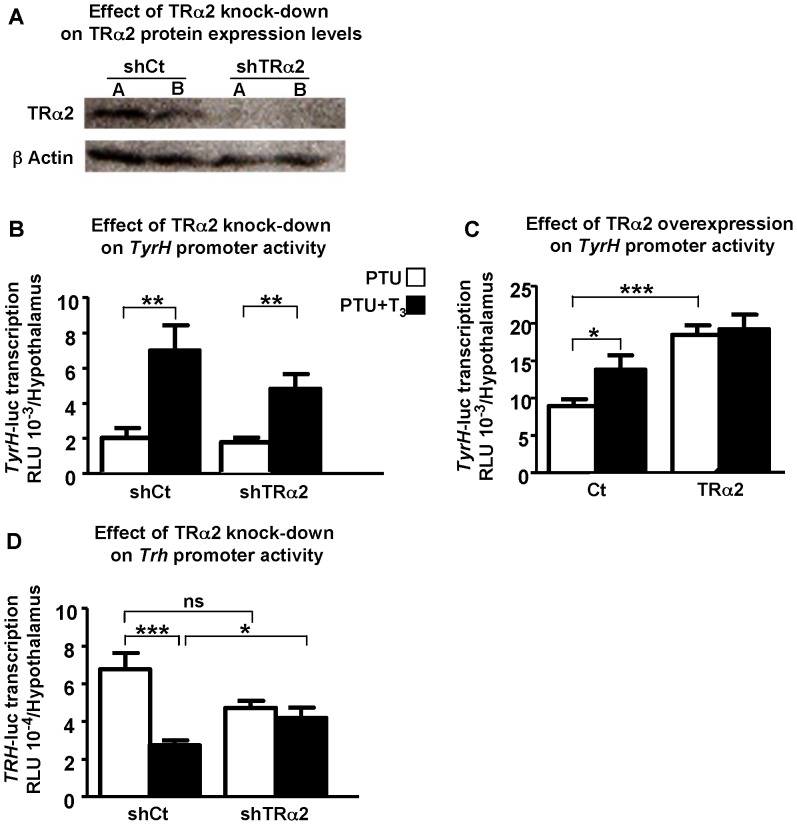
Transcriptional effect of TRα2 knockdown on positively and negatively regulated T_3_ target genes *in vivo*. **A**: **Knockdown of TRα2.** TRα2 expression levels were analyzed on hypothalami (48 h after transfection) of hypothyroid 2-day old mice by western-blot using anti-TRα2 antibody. A decrease in TRα2 protein expression level is observed with shTRα2 compared to control (shCt). Pups were transfected in the hypothalami with 400 ng/pup of shCt (empty pCMV-H1 vector (shCt)) or shTRα2 (mixture of 200 ng pCMV-H1-sh1TRα2 and 200 ng pCMV H1-sh2TRα2 vectors (shTRα2). A and B are samples from different animals. β actin was used as a loading control. **B**: **TRα2 transient knockdown maintains T_3_-dependent activation of the positively regulated** ***TyrH*** **promoter.** ShTRα2 has no effect on *TyrH*-luc transcriptional activity either in absence or presence of T_3_. shCt or shTRα2 (400 ng as above) were co-transfected with 1 µg of *TyrH-luc* construct/hypothalamus of hypothyroid 2-day old mice treated (PTU+T3) or not (PTU) by T_3_ (2.5 µg/g b.w.). **C**: **TRα2 overexpression abrogates T_3_-independent repression of the positively regulated** ***TyrH*** **promoter.** TRα2 overexpression significantly increases T_3_-independent *TyrH-luc* transcription as compared to Ct (p<0.001), but addition of T_3_ does not increase transcription further. Empty pSG5 vector (Ct) or pSG5-TRα2 (TRα2) was used at 100 ng and co-transfected with 1 µg of *TyrH-luc* construct/hypothalamus of hypothyroid 2-day old mice. **D**: **TRα2 transient knockdown abolishes T_3_-dependent repression of the negatively regulated** ***Trh*** **promoter.** ShTRα2 has no effect on T_3_-independent *Trh* promoter activity (p = 0.07) and when T_3_ is added, *Trh-luc* transcription is not repressed anymore (p<0.05) as compared to shCT. The same experimental conditions as in B were used (400 ng expression vector and 1 µg reporter gene, *Trh-luc* per pup). SEMs are given, n≥10 per point. In each case, the whole experiment was repeated twice giving similar results. *, p<0.05; **, p<0.01; ***, p<0.001.

To examine TRα2 transcriptional effects on T_3_ target genes further, transient knockdown of TRα2 was applied. First, in order to determine if the knockdown in TRα2 expression could trigger a detectable decrease in TRα2 protein level, the TRα2 content of the transfected hypothalami was analysed by Western blotting (Figure 2A). To ensure that equivalent amounts of proteins were blotted in each lane, β-actin levels were determined. We find that the amount of TRα2 protein detected in hypothalami 48 h after shTRα2 injection (mix of two sets of shTRα2, sh1TRα2 and sh2TRα2) was strongly decreased compared to TRα2 levels detected in control group (transfected with shCt). This decrease in TRα2 level demonstrates that the knockdown was efficient 48 h after shRNA injection.

We next investigated the effects of transient TRα2 knockdown on a positively regulated T_3_ target gene, Tyrosine hydroxylase enzyme (*TyrH*) using iGT as described above. We found that in controls transfected with shCt (figure 2B left columns), T_3_ significantly increased *TyrH-luc* transcription (p<0.01). The same transcriptional profile is obtained when shTRα2 is co-transfected, with T_3_ significantly increasing *TyrH-luc* transcription (p<0.01) (figure 2B, right columns). Thus, no effect was seen on T_3_-independent and dependent *TyrH-luc* transcriptional levels when shTRα2 is co-transfected as compared to controls (Figure 2B). Only a significant increased effect on T_3_-independent *TyrH-luc* transcription was obtained (p<0.001) when TRα2 was over-expressed as compared to Ct (Figure 2C). We conclude that TRα2 overexpression abrogates T_3_-dependent transcription on both positively regulated T_3_ target genes tested, *ME* as previously mentioned (Figure 1A) and *TyrH* (Figure 2C), whereas TRα2 transient knockdown maintains T_3_-dependent *TyrH-luc* transcription (Figure 2B, far right histograms).

The effects of TRα2 knockdown on the negatively regulated *Trh* gene were examined, using the same *i*GT paradigm with the mix of shTRα2 constructs. In control mice, expression from the *Trh-luc* construct (co-transfected with shCt) was repressed significantly by 60.5% (p<0.001) in animals injected with T_3_ as compared with animals receiving saline (left columns in figure 2D). When the shTRα2 was co-transfected, it abolished the physiological T_3_ regulation of *Trh* (right columns in figure 2D). Transcriptional activity was equivalent as in the group injected with shCt in absence of T_3_, and was unchanged whereas T_3_ was present or not. Thus, loss of TRα2 function seems to allow TRα1 effect on *Trh* promoter being unmasked, resulting in about the same *Trh* promoter activity than when TRα1 is over-expressed. We conclude that, both gain or loss of TRα2 function seems to block *Trh* transcription at an intermediate level between activated and repressed control levels. Indeed, an average TRH activity remains, whereas fine physiological T_3_ regulation is lost.

To test this hypothesis, we next investigated the consequences of gain or loss of TRα2 or TRα1 function on endogenous TRH production.

### Effects of TRα2 or TRα1 gain or loss of function on endogenous TRH production in euthyroid mice

**Figure 3:**
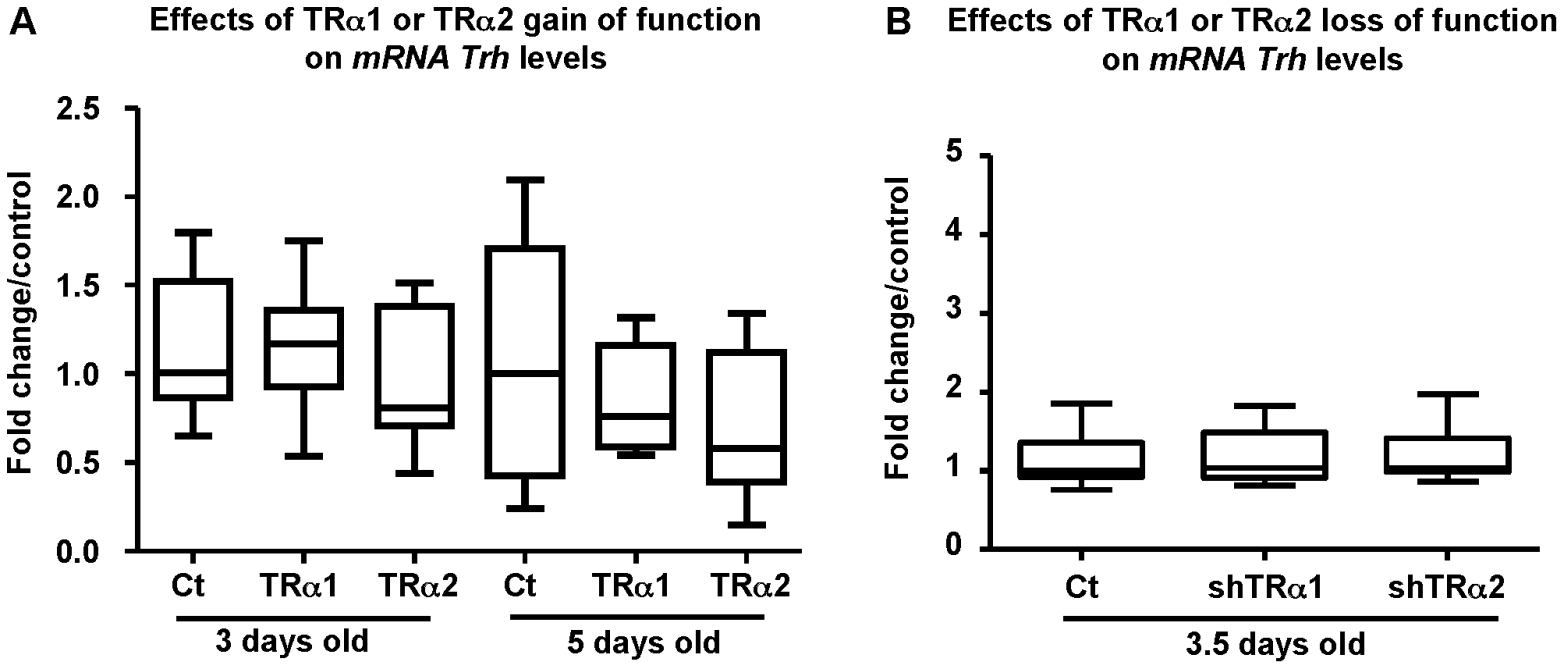
TRα2 or TRα1 gain or loss of function held endogenous *Trh* levels constant. **A**: **TRα2 or TRα1 overexpression does not interfere with endogenous** ***Trh*** **mRNA levels.** Euthyroid 2-days old mice were transfected with 100 ng of empty pSG5 (ct), pSG5-TRα1 (TRα1) or pSG5-TRα2 (TRα2). Hypothalami were dissected at either 3 (3 days old) or 5 (5 days old) days of age (corresponding to 1 day and 3 days post transfection, respectively). Two µg of totRNA were reverse-transcribed and qPCR were performed. Endogenous *Trh* mRNA levels are not significantly modified by TRα2 or TRα1 overexpression in either 3 or 5-day old mice as compared to controls (Ct) *18S* mRNA were used as endogenous control. The whole experiment was repeated twice. **B**: **TRα2 or TRα1 knockdown does not interfere with endogenous** ***Trh*** **mRNA levels.** Euthyroid 2-days old mice were transfected with 400 ng of empty pCMV-H1 (Ct), pCMV-H1-TRα1 (shTRα1) or a mixture of 200 ng sh1TRα2 and 200 ng sh2TRα2 vectors (shTRα2). Hypothalami were dissected 1.5 days post transfection (3.5 days old mice). Endogenous *Trh* mRNA levels are not significantly modified by TRα2 or TRα1 knockdown as compared to control. *Gapdh* mRNA was used as an endogenous control. The whole experiment was repeated twice.

First, TRα1 or TRα2 or a control vector were transfected into the hypothalamus of newborn euthyroid mice. mRNA were extracted and endogenous *Trh* levels were followed by qPCR. As seen in figure 3A, the results show that mRNA *Trh* levels were not significantly modified in either 3 or 5-days old mice when TRα2 or TRα1 was overexpressed, as compared to controls (taken at the same ages). Similarly, no significant effects were seen on endogenous *Trh* levels when shTRα2 or shTRα1 was transfected as compared to controls (Figure 3B). Thus, these results arise the question of determining if at later time points (as compared to shorter times; 1day post transfection corresponding to 3 days old mice), we could see a differential effect of TRα2 or TRα1 on thyroid hormone circulating levels.

### The effects of TRα2 or TRα1 overexpression on *Trh-luc* transcription are correlated with modifications of thyroidal status

**Figure 4:**
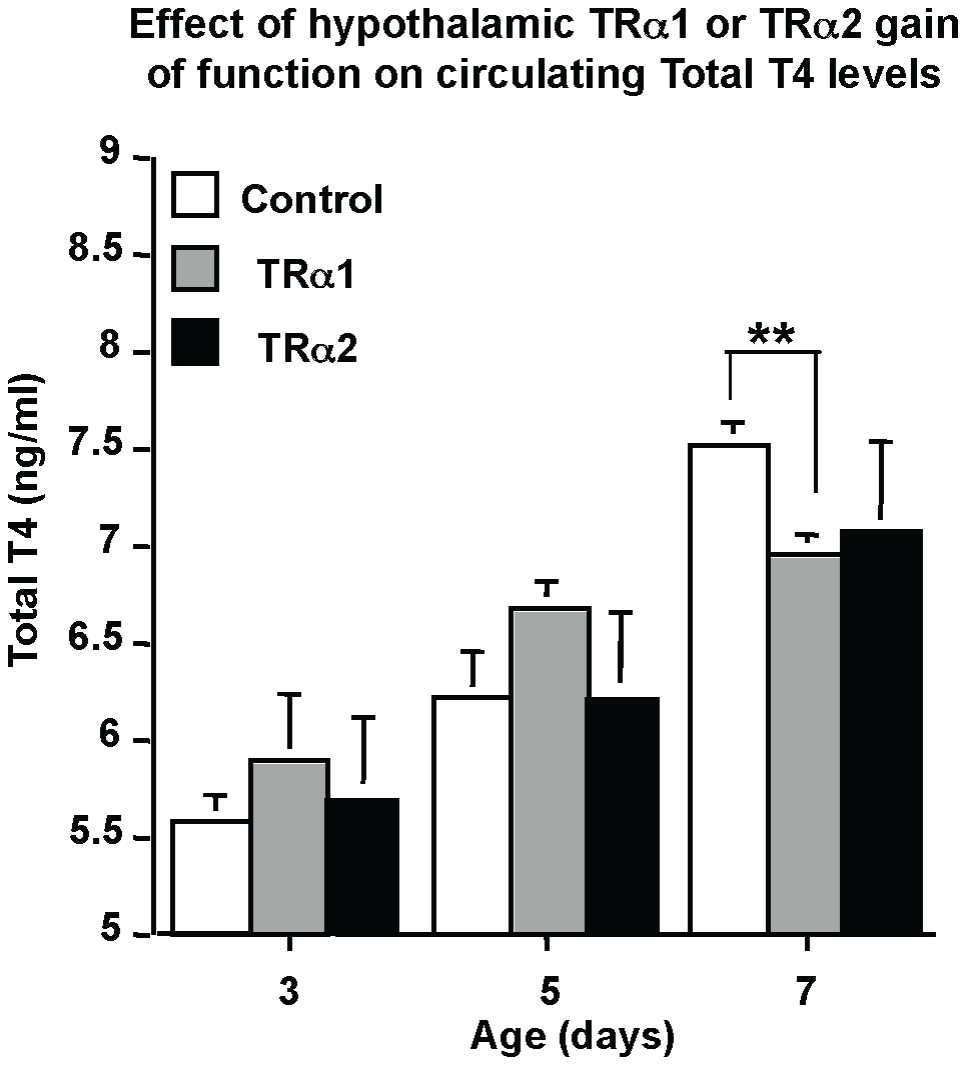
Overexpression of TRα1, but not TRα2, in the hypothalamus modulates circulating T_4_ levels. pSG5-TRα1 (TRα1) or pSG5-TRα2 (TRα2) were transfected (100 ng/pup) into the hypothalamus of hypothyroid 2-day old mice. Serum was collected at the ages shown (3, 5 or 7 days old) and pooled (four individual samples per pool). At 5 days post-transfection (7 days old mice), T_4_ circulating levels are significantly decreased when TRα1 is overexpressed and not modified by TRα2 overexpression. Means ± SEM of pooled samples are given, n≥4 for each point. **, p<0.01.

Given the differential effects of TRα2 versus TRα1 on the *Trh* promoter activity obtained by iGT, we next examined the effects of their overexpression on circulating T_4_ levels. As seen in figure 4, TRα1 overexpression resulted in a significantly decreased circulating T_4_ level at P7 as compared to controls (p<0.01) (Figure 4, far right columns). However no effect in circulating T_4_ was observed at the same age when TRα2 was overexpressed. The results of TRα2 or TRα1 overexpression on *Trh-luc* transcription are correlated with modifications of thyroidal status.

## Discussion

It is intriguing to note that of all the four main products (TRα1, TRα2, TRβ1 and TRβ2) of the two TR genes (NR1A1 and NR1A2), the mRNA of the non-hormone-binding variant TRα2 is by far the most highly expressed in the brain [6], [26]. Indeed in the rat brain, temporal expression of TRα2 mRNA follows the same spatial pattern of expression of TRα1, but its levels are markedly higher [26], suggesting that TRα2 might be a critical non T_3_ dependent regulator of thyroid hormone action by modulating T_3_-binding TR effects on the expression of brain-specific genes [7]. One line of investigation to address the role of TRα2 in general thyroid hormone dependent signalling has been to generate mice lacking TRα2. These mice show an overexpression of TRα1 in all tissues examined, including brain. The mice have significantly lower circulating free T_3_ and free T_4_ and their thyroid glands show features of dysfunction, suggesting decreased activity of the Hypothalamic Pituitary Thyroid axis (H-P-T) [20]. This phenotype (insufficient stimulation of the thyroid and of the production of TH) raises the question of the physiological function of TRα2 in brain and notably in the hypothalamus at the level of *Trh* transcription.

We chose to examine the effects of TRα2 on *Trh* transcription using an *in vivo* reporter gene approach. Three reasons, besides the phenotype of the TRα2^-/-^ mouse, made *Trh* promoter of a particular interest in terms of function of this enigmatic TRα2 isoform. First, *Trh* gene regulation allows one to investigate TR isoforms specificity as TRβ and TRα have distinct roles in the negative transcriptional regulation by T_3_ [1], [27]. Second, *Trh* is a critical component of the H-P-T axis and is thus a critical regulatory gene. Third, *Trh* is a T_3_ negatively regulated target gene and is of particular mechanistic interest from the transcriptional point of view. It is important to discuss here the fact that it is often considered that the HPT axis is immature in the postnatal mouse. This concept is largely based on the observations of the low levels of circulating T_3_ and T_4_ levels that increase steadily during the first two weeks of postnatal life peaking at p15 and then declining slightly to reach adult levels [28]. However, the feedback system is active as decreasing T_3_ and T_4_ levels by administrating PTU increase *Trh* expression. Thus even if the axis is not fully mature, the components of negative feedback are present (TRs, NCoR, SMRT, etc…[29]). In fact, just because circulating levels of T_3_ and T_4_ climb during this post-natal phase does not actually imply that the axis is not functional until adult levels are attained. First, the low levels of circulating hormone indicate more that is could be due to low feed forward drive at any of the levels, TRH, TSH or even T_3_/T_4_ production. Second, these low levels do not rule out the possibility that the feedback system can respond to high levels of T_3_. Thus more knowledge is required on the manner at which hypothalamic setpoints are established, and modulated, during this critical post-natal period.

We started our study by iGT experiments conducted on hypothyroid newborn mice, to reduce high variability in endogenous thyroid hormone levels, which could compromise transcriptional regulation study. The results on *Trh* transcription were compared to those obtained on *Malic Enzyme (ME)*. In both cases we compared the effects of TRα2 to those obtained with TRα1, because TRα1 action on both genes of interest has already been well characterised *in vivo*. We observed that in absence of T_3_, TRα1 fails to repress *ME* expression, suggesting that level of endogenous TRα1 was already sufficient to repress basal *ME* expression. In contrast, TRα2 was able to increase basal *ME* transcription, suggesting that when TRα2 is overexpressed it acts as a dominant-negative receptor, competing with endogenous TRα1 as to lead to an increase in *ME* transcription.

Regarding the negatively T_3_-regulated *Trh* gene, TRα1 prevents the T_3_-indepedent *Trh* activation, and increases the T_3_-dependent repression observed in the control group. Thus, TRα2 acts as a dominant-negative receptor on both positively and negatively regulated T_3_ target genes. Our *in vivo* result on *ME-tk-luc* transcription was in accordance with data conducted on transfected cells where TRα2 exerted a negative effect on T_3_-positive response element-mediated transcription [30]. The molecular mechanisms underlying the dominant negative activity of TRα2 are not yet completely elucidated, even *in vitro*. Two different mechanisms have been proposed: the first, described by Katz et al. [15] involves a passive repression, in which TRα2 blocks TRs action by competing for binding to TREs; the second mechanism has been proposed by Liu et al. [17], who demonstrated that TRα2 inhibitory effect does not require binding to TRE and suggested that interactions with components of the general transcription machinery might instead play a crucial role.

For the T_3_ negatively regulated gene *Trh*, the gain of function of TRα2, when compared to the gain of function of TRα1, results in strong activation of transcription that is unmodified by presence or absence of T_3_. In contrast, TRα1 overexpression down-regulates *Trh* transcription and this regulation is equally T_3_ insensitive. Each of these regulations, contrast with the physiological T_3_-dependent repression of *Trh* in the presence of TRβ isoforms [2], [21]. This T_3_-independent activation of *Trh* transcription by TRα2 suggests a possible role of TRα2 *in vivo,* acting as a dominant-negative receptor on negative T_3_-regulated genes. *In vitro* experiments have not been able to reveal such a role. When transfected into JEG-3 cells, TRα2 isoform was inactive on positively and negatively regulated T_3_ response genes whereas TRα1 and TRβ stimulated transcription from *TRE-tk-CAT* (pTRE), and repressed *TSHα-CAT* (nTRE) reporter genes in T_3_-dependent manners. When coexpressed with TRα1 or TRβ at relatively high doses, TRα2 inhibited regulation of positive TREs but did not affect negative regulation [14]. The difference between these findings and our data are probably due to different cellular contexts and different target genes studied.

To explore further the function of TRα2 in *Trh* regulation, we used transient knockdown of TRα2 using an shRNA approach. Effects were also followed on positively T_3_-regulated target genes (Tyrosine hydroxylase (*TyrH*) and *ME* enzymes). TRα2 overexpression abrogated T_3_-dependent transcription on both of these positively T_3_ regulated target genes, whereas TRα2 transient knockdown maintains T_3_-dependent *TyrH-luc* transcription. This result confirms the dominant negative action of TRα2 on positively regulated T_3_ target genes since its knockdown unmasks functional TRs transcriptional effects. Intriguingly, transient knockdown of TRα2 has the same effect as its overexpression on *Trh* gene transcription (*Trh-luc* transcriptional levels are similar to those obtained in controls in absence or presence of T_3_). Indeed, when TRα2 is overexpressed it leads to an imbalance in the transcriptional machinery, thus impairing the well-defined effect of TRβ on T_3_-dependent *Trh* transcriptional repression [23], [31]. Conversely, when shTRα2 is transfected, a shift in the balance of the transcriptional machinery towards TRα1 results in equivalent *Trh-luc* transcriptional levels as when TRα1 is overexpressed.

In order to study the effects of TRα1 and TRα2 on endogenous *Trh mRNA* levels, qPCR analyses were conducted on euthyroid mice so as to examine the dynamics of feedback in physiologically normal animals. We obtained no differential effects of the two overexpressed isoforms in the shorter frame at one day post-transfection (3 days old mice) nor at three days post-transfection. Similarly, no detectable effect was seen on endogenous *Trh* mRNA levels in the shorter frame (at 36 h post-transfection) when shTRα2 or shTRα1 was transfected. These data showing no detectable variations in *Trh* mRNA levels in the shorter term fit with those published by a number of authors [32], [33] who showed *Trh* mRNA varied within longer time frames.

To propose a model of TRα1 and TRα2 interaction, it is easiest to start from the TRα2 loss of function studies. The mutant mice show loss of TRα2 to increase TRα1 expression. In effect, we observe that hypothalamic TRα2 loss of function has the same effect on *Trh-luc* transcriptional activity as TRα1 overexpression. We therefore suggest that in physiological conditions there is a balance between the effects of TRα2 and TRα1 allowing the overriding effects of the TRβ isoforms that provide physiological T_3_-dependent *Trh* regulation.

Indeed, the results from the mutant mice studies [20] suggest that the loss of TRα2 or the changed balance of TRα2/TRα1 perturbs a range of functions (metabolism and growth) notably at the central level, suggesting a role for TRα2 in regulating central T_3_-dependent transcription genes. Similarly, a changed balance of TRα2/TRα1 in a context where TRα2 is overexpressed would have consequent transcriptional effects on brain gene expression. A biological activity that can be attributed to TRα2 would be an adjustment of the T_3_ binding TRα1 protein activity to physiologically appropriate levels, implying thus an important, widespread regulatory role in mammalian physiology of the ratio of TRα1/TRα2 expression. Our data showing that TRα2 does indeed act as a dominant-negative receptor on both negatively and positively T_3_ regulated target genes in the brain strongly bolster this hypothesis.

In this report we define an *in vivo* function for the nonbinding TRα2 isoform in regulating brain genes, supported by findings on athyroid Pax8^-/-^ TRα1^-/-^ mice who die around weaning unless they are substituted with thyroid hormones due to the negative effects of the TRα1 aporeceptor, but, rather, including a more complex mechanism involving TRα2 and unliganded TR isoform TRΔα2 [34].

Taken together, our results emphasize the as yet neglected physiological importance of TRα2, naturally acting as dominant-negative receptor on hypothalamic *Trh* transcription, and consequently, on the regulation of HPT axis.
